# Bistable Firing Pattern in a Neural Network Model

**DOI:** 10.3389/fncom.2019.00019

**Published:** 2019-04-05

**Authors:** Paulo R. Protachevicz, Fernando S. Borges, Ewandson L. Lameu, Peng Ji, Kelly C. Iarosz, Alexandre H. Kihara, Ibere L. Caldas, Jose D. Szezech, Murilo S. Baptista, Elbert E. N. Macau, Chris G. Antonopoulos, Antonio M. Batista, Jürgen Kurths

**Affiliations:** ^1^Graduate in Science Program—Physics, State University of Ponta Grossa, Ponta Grossa, Brazil; ^2^Center for Mathematics, Computation, and Cognition, Federal University of ABC, São Bernardo do Campo, Brazil; ^3^National Institute for Space Research, São José dos Campos, Brazil; ^4^Institute of Science and Technology for Brain-Inspired Intelligence, Fudan University, Shanghai, China; ^5^Key Laboratory of Computational Neuroscience and Brain-Inspired Intelligence (Fudan University), Ministry of Education, Shanghai, China; ^6^Institute of Physics, University of São Paulo, São Paulo, Brazil; ^7^Department of Mathematics and Statistics, State University of Ponta Grossa, Ponta Grossa, Brazil; ^8^Institute for Complex Systems and Mathematical Biology, SUPA, University of Aberdeen, Aberdeen, United Kingdom; ^9^Department of Mathematical Sciences, University of Essex, Colchester, United Kingdom; ^10^Potsdam Institute for Climate Impact Research, Potsdam, Germany; ^11^Department of Physics, Humboldt University, Berlin, Germany

**Keywords:** bistable regime, network, adaptive exponential integrate-and-fire neural model, neural dynamics, synchronization, epilepsy

## Abstract

Excessively high, neural synchronization has been associated with epileptic seizures, one of the most common brain diseases worldwide. A better understanding of neural synchronization mechanisms can thus help control or even treat epilepsy. In this paper, we study neural synchronization in a random network where nodes are neurons with excitatory and inhibitory synapses, and neural activity for each node is provided by the adaptive exponential integrate-and-fire model. In this framework, we verify that the decrease in the influence of inhibition can generate synchronization originating from a pattern of desynchronized spikes. The transition from desynchronous spikes to synchronous bursts of activity, induced by varying the synaptic coupling, emerges in a hysteresis loop due to bistability where abnormal (excessively high synchronous) regimes exist. We verify that, for parameters in the bistability regime, a square current pulse can trigger excessively high (abnormal) synchronization, a process that can reproduce features of epileptic seizures. Then, we show that it is possible to suppress such abnormal synchronization by applying a small-amplitude external current on > 10% of the neurons in the network. Our results demonstrate that external electrical stimulation not only can trigger synchronous behavior, but more importantly, it can be used as a means to reduce abnormal synchronization and thus, control or treat effectively epileptic seizures.

## 1. Introduction

Epilepsy is a brain disease that causes seizures and sometimes loss of consciousness (Chen et al., 2014, 2015). Epileptic seizures are associated with excessively high synchronous activities (Li et al., 2007; Jiruska et al., 2013; Wu et al., 2015) of neocortex regions or other neural populations (Fisher et al., 2005; Sierra-Paredes and Sierra-Marcuño, 2007; Engel et al., 2013; Geier and Lehnertz, 2017; Falco-Walter et al., 2018). Electroencephalography has been used to identify and classify seizures (Noachtar and Rémi, 2009), as well as to understand epileptic seizures (Scharfman and Buckmaster, 2014). Abnormal activities have a short period of time, lasting from a few seconds to minutes (Trinka et al., 2015), and they can occur in small or larger regions in the brain (McCandless, 2012; Kramer and Cash, 2012). Two suggested mechanisms responsible for the generation of partial epilepsy are the decrease of inhibition and increase of excitation (McCandless, 2012). In experiments and simulations, the reduction of excitatory and the increase of inhibitory influence have been effective in suppressing and preventing synchronized behaviors (Traub et al., 1993; Schindler et al., 2008). Traub and Wong (1982) showed that synchronized bursts that appear in epileptic seizures depend on neural dynamics.

Single seizures can not kill neurons, however recurrent ones can do so and thus, can lead to chronic epilepsy (Dingledine et al., 2014). Evidence that supports this further is provided by abnormal anatomical alterations, such as mossy fiber sprouting (Danzer, 2017), dendritic reconfigurations (Wong, 2005, 2008), and neurogenesis (Jessberger and Parent, 2015; Cho et al., 2015). In fact, such alterations change the balance between inhibition and excitation (Holt and Netoff, 2013; Silva et al., 2003). Wang et al. (2017) demonstrated that a small alteration in the network topology can induce a bistable state with an abrupt transition to synchronization. Some *in vitro* seizures generated epileptiform activities when inhibitory synapses were blocked or excitatory synapses were enhanced (Traub et al., 1994; White, 2002). Several studies showed that epileptiform activities are related not only with unbalanced neural networks, but also with highly synchronous regimes (Uhlhaas and Singer, 2006; Andres-Mach and Adamu, 2017).

Different routes to epileptic seizures were reported by Silva et al. (2003). The authors considered epilepsy as a dynamical disease and presented a theoretical framework where epileptic seizures occur in neural networks that exhibit bistable dynamics. In the bistable state, transitions can happen between desynchronous and synchronous behaviors. Suffczynski et al. (2004) modeled the dynamics of epileptic phenomena by means of a bistable network.

Many works reported that periodic electrical pulse stimulation facilitates synchronization, while random stimulation promotes desynchronization in networks (Cota et al., 2009). Electrical stimulation can be applied in different brain areas, for instance in the hippocampus, thalamus, and cerebellum (McCandless, 2012). The mechanism for electrical stimulation to cease seizures is still not completely understood, however, signal parameters such as frequency, duration, and amplitude can be changed to improve the efficiency of the treatment of epilepsy (McCandless, 2012). The electrical stimulation has been used as an efficient treatment for epilepsy in the hippocampus (Velasco et al., 2007). In Antonopoulos (2016), the author studied external electrical perturbations and their responses in the brain dynamic network of the Caenorhabditis elegans soil worm. It was shown that when one perturbs specific communities, keeping the others unperturbed, the external stimulations propagate to some but not all of them. It was also found that there are perturbations that do not trigger any response at all and that this depends on the initially perturbed community.

Neural network models have been used to mimic phenomena related to neural activities in the brain. Guo et al. (2016a) built a network model where the postsynaptic neuron receives input from excitatory presynaptic neurons. They incorporated autaptic coupling (Guo et al., 2016b) in a biophysical model. Delayed models have been considered in biological systems (Khajanchi et al., 2018), for instance, Sun et al. (2018) analyzed the influence of time delay in neuronal networks. They showed that intra- and inter-time delays can induce fast regular firings in clustered networks. In this work, we build a random network with neural dynamics to study synchronization induced in a bistable state which is related to epileptic seizures. In particular, we consider a network composed of adaptive exponential integrate-and-fire (AEIF) neurons coupled by means of inhibitory and excitatory synapses. The AEIF model mimics phenomenological behaviors of neurons (Clopath et al., 2006) and is appropriate to study even large networks (Naud et al., 2008). Borges et al. (2017) verified that depending on the excitatory synaptic strength and connection probability, a random network of coupled AEIF neurons can exhibit transitions between desynchronized spikes and synchronized bursts (Protachevicz et al., 2018). In the network considered here, we observe the existence of bistability when it is unbalanced, namely that the decrease of synaptic inhibition induces a bistable state. We analyse the effects of the application of external square current pulses (SCP) by perturbing the neural dynamics on the network using parameters that lead to a bistable state, such as the excitatory and inhibitory synaptic conductances. We find that, depending on the duration and amplitude of the external current, SCP can either trigger or suppress synchronization in the bistability region, an idea that can be used further to treat epilepsy by suppressing excessive synchronization in affected brain regions.

## 2. Methods

### 2.1. Neural Network Model

We build a random network of *N* = 1, 000 adaptive exponential integrate-and-fire neurons (Brette and Gerstner, 2005) with probability *p* for the formation of connections among them equal to 0.1. The network consists of 80% excitatory and 20% inhibitory neurons (Noback et al., 2005). The dynamics of each neuron *i, i* = 1, …, *N* in the network is given by the set of equations

(1) $$\begin{array}{rcl} C_{\text{m}}\frac{dV_{i}}{dt} & = & -g_{L}\left(V_{i}-E_{L}\right)+g_{L}\Delta_{T}\exp\left(\frac{V_{i}-V_{T}}{\Delta_{T}}\right)\\ + & I_{i}-w_{i}+\overset{N}{\underset{j=1}{\sum }}\left(V_{\text{REV}}^{j}-V_{i}\right)M_{ij}g_{j}+\Gamma_{i},\\ \tau_{w}\frac{dw_{i}}{dt} & = & a_{i}\left(V_{i}-E_{L}\right)-w_{i},\\ \tau_{s}\frac{dg_{i}}{dt} & = & -g_{i}. \end{array}$$

The membrane potential *V*_*i*_ and adaptation current *w*_*i*_ represent the state of each neuron *i*. The capacitance membrane *C*_m_ is set to *C*_m_ = 200 pF, the leak conductance to *g*_*L*_ = 12 nS, the resting potential to *E*_*L*_ = −70 mV, the slope factor to Δ_*T*_ = 2.0 mV and the spike threshold to *V*_*T*_ = −50 mV. The adaptation current depends on the adaptation time constant τ_*w*_ = 300 ms and the level of subthreshold adaptation *a*_*i*_ that is randomly distributed in the interval [0.19, 0.21] nS. We consider the injection of current *I*_*i*_ to each neuron *i* in terms of the relative rheobase current *r*_*i*_ = *I*_*i*_/*I*_rheobase_ (Naud et al., 2008). The rheobase is the minimum amplitude of the applied current to generate a single or successive firings. The application of this constant current allows neurons to change their potentials from resting potentials to spikes. The value of the rheobase depends on the neuron parameters. The external current arriving at neuron *i* is represented by Γ_*i*_. We consider the external current according to a SCP with amplitude *A*_*I*_ and time duration *T*_*I*_. The random connections in the network are described by the binary adjacency matrix *M*_*ij*_ with entries either equal to 1 when there is a connection from *i* to *j* or 0 in the absence of such a connection. *g*_*i*_ is the synaptic conductance, τ_*s*_ the synaptic time constant, and *V*_REV_ the synaptic reversal potential. We consider τ_*s*_ = 2.728 ms, *V*_REV_ = 0mV for excitatory synapses, and *V*_REV_ = −80 mV for inhibitory synapses. The synaptic conductance decays exponential with a synaptic time constant τ_*s*_. When the membrane potential of neuron *i* is above the threshold *V*_*i*_ > *V*_thres_ (Naud et al., 2008), the state variable is updated by the rule

(2) $$\begin{array}{rcl} V_{i} & \to & V_{r}=-58\text{mV},\\ w_{i} & \to & w_{i}+70\text{pA},\\ g_{i} & \to & g_{i}+g_{s}, \end{array}$$

where *g*_*s*_ assumes the value of *g*_exc_ when neuron *i* is excitatory (*i* ≤ 0.8*N*) and *g*_inh_ when neuron *i* is inhibitory (*i* > 0.8*N*). In this work, we study the parameter space (*g*_exc_, *g*_inh_) and consider a relative inhibitory synaptic conductance *g* = *g*_inh_/*g*_exc_. We consider parameter values in which the individual uncoupled neurons perform spike activities. The initial values of *V* and *w* are randomly distributed in the interval [−70, −50] mV and [0, 70] pA, respectively. The initial *g*_*i*_ value is equal to 0.

### 2.2. Synchronization

The synchronous behavior in the network can be identified by means of the complex phase order parameter (Kuramoto, 1984)

(3) $$\begin{array}{r} R\left(t\right)\exp\left(\text{i}\Phi \left(t\right)\right)\equiv \frac{1}{N}\overset{N}{\underset{j=1}{\sum }}\exp\left(\text{i}\psi_{j}\left(t\right)\right), \end{array}$$

where *R*(*t*) and Φ(*t*) are the amplitude and angle of a centroid phase vector over time, respectively. The phase of neuron *j* is obtained by means of

(4) $$\begin{array}{r} \psi_{j}\left(t\right)=2\pi m+2\pi \frac{t-t_{j,m}}{t_{j,m+1}-t_{j,m}}, \end{array}$$

where *t*_*j, m*_ corresponds to the time of the *m*−th spike of neuron *j* (*t*_*j, m*_ < *t* < *t*_*j, m*+1_) (Rosenblum et al., 1996, 1997). We consider that the spike occurs for *V*_*j*_ > *V*_thres_. *R*(*t*) is equal to 0 for fully desynchronized and 1 for fully synchronized patterns, respectively.

We calculate the time-average order parameter $\bar{R}$ (Batista et al., 2017) given by

(5) $$\begin{array}{r} \bar{R}=\frac{1}{t_{\text{fin}}-t_{\text{ini}}}\int_{t_{\text{ini}}}^{t_{\text{fin}}}R\left(t\right)dt, \end{array}$$

where *t*_fin_ − *t*_ini_ is the time window. We consider *t*_fin_ = 200s and *t*_ini_ = 180s.

### 2.3. Synaptic Input

We monitor the instantaneous synaptic conductances arriving at each neuron *i* through

(6) $$\begin{array}{r} I_{i}^{\text{ISC}}\left(t\right)=\overset{N}{\underset{j=1}{\sum }}\left(V_{\text{REV}}^{j}-V_{i}\right)M_{ij}g_{j}. \end{array}$$

The instantaneous synaptic input changes over time due to the excitatory and inhibitory inputs received by neuron *i*. The average instantaneous synaptic conductances is given by

(7) $$\begin{array}{r} I_{\text{syn}}\left(t\right)=\frac{1}{N}\overset{N}{\underset{i=1}{\sum }}I_{i}^{\text{ISC}}\left(t\right). \end{array}$$

### 2.4. Coefficient of Variation

The *m*−th inter-spike interval ${\text{ISI}}_{i}^{m}$ is defined as the difference between two consecutive spikes of neuron *i*,

(8) $$\begin{array}{r} {\text{ISI}}_{i}^{m}=t_{i}^{m+1}-t_{i}^{m}>0, \end{array}$$

where $t_{i}^{m}$ is the time of the *m*−th spike of neuron *i*.

Using the mean value of ISI_*i*_, ${\bar{\text{ISI}}}_{i}$, and its standard deviation, σ_ISI_*i*__, we calculate the coefficient of variation (CV)

(9) $$\begin{array}{r} {\text{CV}}_{i}=\frac{\sigma_{{\text{ISI}}_{i}}}{{\bar{\text{ISI}}}_{i}}. \end{array}$$

The average of CV ($\bar{\text{CV}}$) is then obtained through

(10) $$\begin{array}{r} \bar{\text{CV}}=\frac{1}{N}\overset{N}{\underset{i=1}{\sum }}{\text{CV}}_{i}. \end{array}$$

Finally, we use $\bar{\text{CV}}$ to identify spike (when $\bar{\text{CV}}<0.5$) and burst fire patterns (when $\bar{\text{CV}}\geq 0.5$) (Borges et al., 2017; Protachevicz et al., 2018).

### 2.5. Instantaneous and Mean Firing-Rate

The instantaneous firing-rate in intervals of *t*_step_ = 1ms is given by

(11) $$\begin{array}{r} F\left(t\right)=\frac{1}{N}\overset{N}{\underset{i=1}{\sum }}\left(\int_{t}^{t+t_{\text{step}}}\delta \left(t^{\prime }-t_{i}\right)dt^{\prime }\right), \end{array}$$

where *t*_*i*_ is the firing time of neuron *i* in the time interval (*t* ≤ *t*_*i*_ ≤ *t* + 1) ms. This measure allows to obtain the instantaneous population activity in the network. The mean firing-rate can then be calculated by means of

(12) $$\begin{array}{r} \bar{F}=\frac{1}{\bar{\text{ISI}}}, \end{array}$$

where $\bar{\text{ISI}}$ is the average ISI obtained over all *N* neurons in the network, that is $\bar{\text{ISI}}=\frac{1}{N}\overset{N}{\underset{i=1}{\sum }}{\bar{\text{ISI}}}_{i}$.

## 3. Results

### 3.1. Inhibitory Effect on Synchronous Behavior

**Figure 1:**
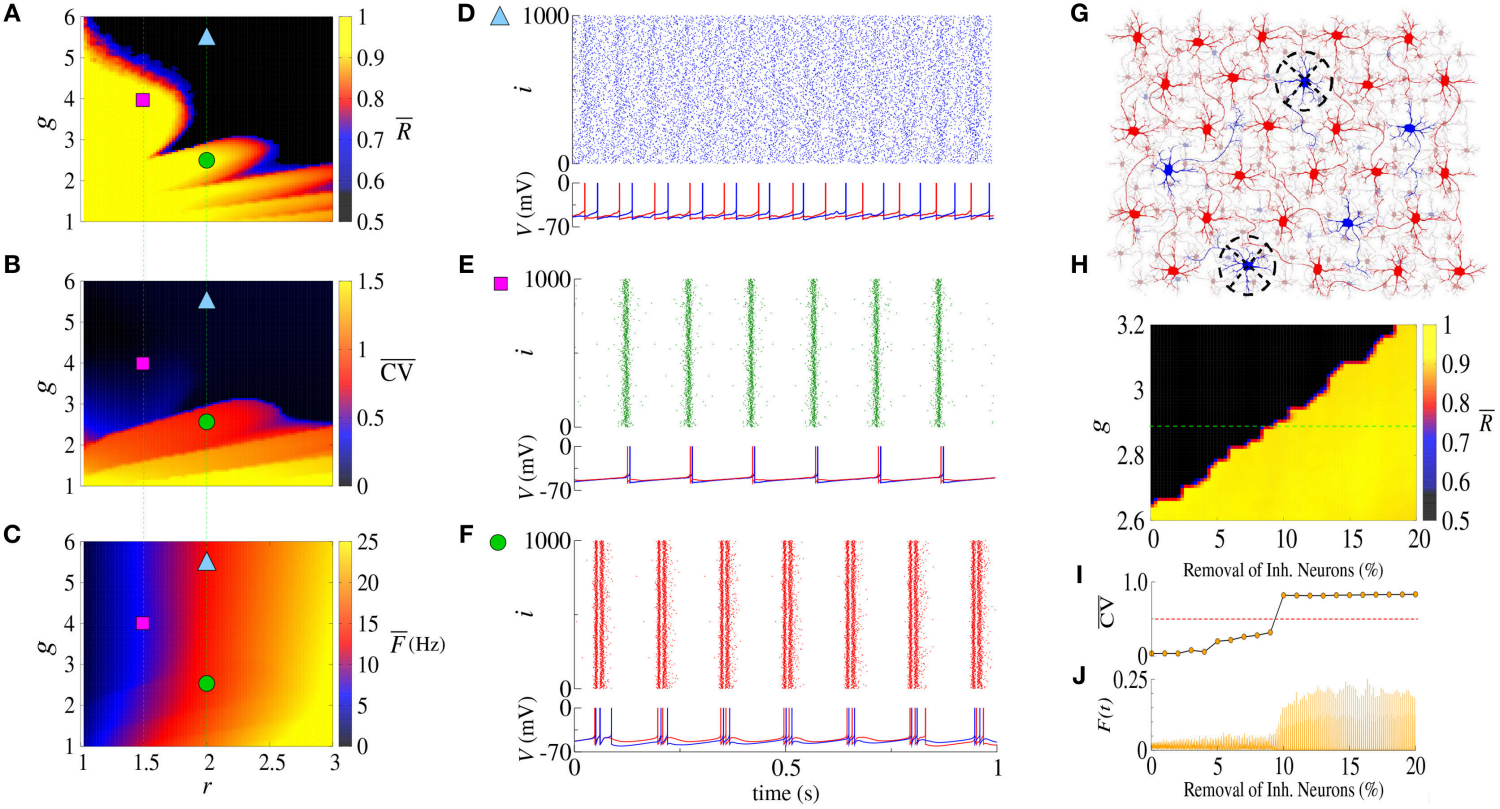
Parameter space (*g, r*) for the **(A)** time-average order parameter ($\bar{R}$), **(B)** the mean coefficient of variation ($\bar{\text{CV}}$), and **(C)** the mean firing-rate ($\bar{F}$). Raster plot that displays the spiking activity over time and membrane potential are shown for **(D)** desynchronized spikes for *r* = 2.0 and *g* = 5.5 (cyan triangle), **(E)** synchronized spikes for *r* = 1.5 and *g* = 4 (magenta square), and **(F)** synchronized bursts for *r* = 2.0 and *g* = 2.5 (green circle). Here, we consider *g*_exc_ = 0.4nS. In **(G)**, we illustrate a network composed of excitatory (red) and inhibitory (blue) neurons, where some inhibitory neurons are removed (black dashed circle). **(H)** Shows the time-average order parameter for *g* vs. the percentage of inhibitory neurons removed from the network. The green dashed line corresponds to *g* = 2.9. The values of $\bar{\text{CV}}$ and instantaneous firing-rate are shown in **(I,J)**, respectively.

The balance between excitation and inhibition generates an asynchronous activity in the network (Lundqvist et al., 2010; Ostojic, 2014). However, for the unbalanced network we observe synchronized spikes and bursts. Figures 1A–C show the time-average order parameter ($\bar{R}$), the mean coefficient of variation ($\bar{\text{CV}}$) and the mean firing-rate ($\bar{F}$), respectively, for the parameter space (*g, r*), where *g* is the ratio between inhibitory (*g*_inh_) and excitatory (*g*_exc_) synaptic conductances, and *r* the relative rheobase current. For *g*_exc_ = 0.4nS and *g* > 6, we observe that $\bar{R}<0.5$ and that $\bar{\text{CV}}<0.5$, corresponding to desynchronized spikes. In Figure 1D, we see the raster plot and membrane potential for 2 neurons in the network with a desynchronized spike-pattern for *g* = 5.5 and *r* = 2 (blue triangle). For *g* = 4 and *r* = 1.5 (magenta square), the dynamics exhibits synchronized spikes (Figure 1E), as a result of setting $\bar{R}>0.9$ and $\bar{\text{CV}}<0.5$. Figure 1F shows synchronized bursts of activity for *g* = 2.5 and *r* = 2 (green circle), where $\bar{R}>0.9$ and $\bar{\text{CV}}\geq 0.5$. Within this framework, we have verified the existence of transitions from desynchronized spikes to synchronized bursting activities without significant changes in the mean firing-rate.

The appearance of synchronous behavior cannot only be related to the decrease of the inhibitory synaptic strength, but also to a loss of inhibitory neurons. In particular, we show this in Figure 1G which illustrates a network composed of excitatory (red) and inhibitory (blue) neurons, where some inhibitory neurons were removed (dashed circles). In Figure 1H, we see that the synchronous behavior depends on *g* and the percentage of removed inhibitory neurons. Figure 1I shows the transition from spiking dynamics ($\bar{\text{CV}}<0.5$) to bursting dynamics ($\bar{\text{CV}}\geq 0.5$), and Figure 1J shows the instantaneous firing-rate *F*(*t*). For *g* = 2.9 and *g*_exc_ = 0.4nS (green dashed line), the transition to synchronized bursts occurs when 10% of inhibitory neurons are removed from the network, and as a consequence *F*(*t*) reaches the maximum value of 0.2.

Concluding, alterations in the inhibitory synaptic strength or in the number of inhibitory neurons can induce transition to synchronous patterns. Wang et al. (2017) presented results where synchronization transition occurs as a result of small changes in the topology of the network, whereas here, we study transitions caused due to changes in the inhibitory synaptic strength and the emergence of a bistable regime.

### 3.2. Bistable Regime

**Figure 2:**
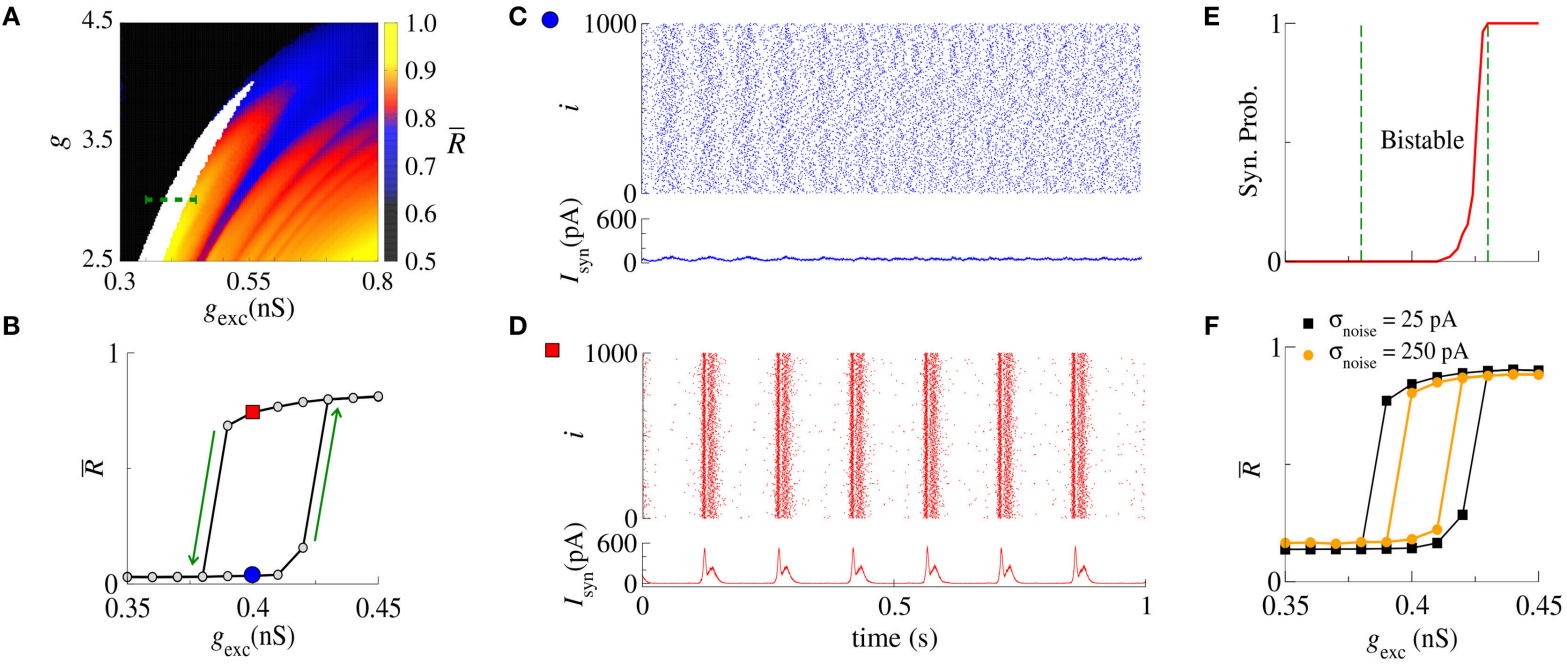
**(A)** The parameter space (*g, g*_exc_) for *r* = 2, where $\bar{R}$ is encoded in color. The black region corresponds to desynchronized activity, whereas colored regions indicate $\bar{R}>0.6$ and the white region represents the bistable regime. **(B)** The bistable region indicated in the parameter space of **(A)** by means of a green dashed line. **(C,D)** Show the raster plots and *I*_syn_ for desynchronized spikes (blue circle) and synchronized bursts (red square), respectively. We identify bistability by checking when ${\bar{R}}_{\text{backward}}-{\bar{R}}_{\text{forward}}>0.4$ and consider two trials for each set of parameter values. **(E)** The synchronization probability as a function of *g*_exc_. **(F)** $\bar{R}\times g_{\text{exc}}$ for σ_noise_ equal to 25 pA and 250 pA.

Next, we analyse synchronization in the parameter space (*g, g*_exc_). In particular, Figure 2A shows $\bar{R}$ with values depicted in the color bar. The black region corresponds to desynchronized spike activity, while the remaining colored regions are associated with burst activities. The white region represents the bistable regime, where desynchronized spikes or synchronized bursts are possible depending on the initial conditions. In the bistable regime, decreasing *g*_exc_ (backward direction), $\bar{R}$ is higher than increasing *g*_exc_ (forward direction), as shown in Figure 2B for *g* = 3, *r* = 2, and *g*_exc_ = [0.35, 0.45] nS (green dashed line in Figure 2A). We identify bistability (white region) in the parameter space when the condition ${\bar{R}}_{\text{backward}}-{\bar{R}}_{\text{forward}}>0.4$ is fulfilled. The raster plot and instantaneous synaptic input for desynchronized spikes (blue circle) and synchronized bursts (red square) are shown in Figures 2C,D, respectively. When the dynamics on the random network is characterized by desynchronized spikes, the instantaneous synaptic inputs exhibit *I*_syn_(*t*) ≈ 50pA. For synchronized bursts, *I*_syn_(*t*) ≈ 0 when a large number of neurons in the network are silent (i.e., not firing), and *I*_syn_(*t*) > 200 pA during synchronous firing activities. In Figure 2E, we compute the probability of occurrence of excessively high synchronicity within the bistable regime. We observe a small synchronization probability value in the bistable region. This result has a biological importance due to the fact that the seizure state is a relatively small probability event compared with the normal state. DaQing et al. (2017) showed that noise can regulate seizure dynamics in partial epilepsy. Figure 2F displays $\bar{R}\times g_{\text{exc}}$ for Gaussian noise with mean 0 and standard deviation σ_noise_ equal to 25 pA and 250 pA. We verify that the bistable region decreases when the noise level increases.

**Figure 3:**
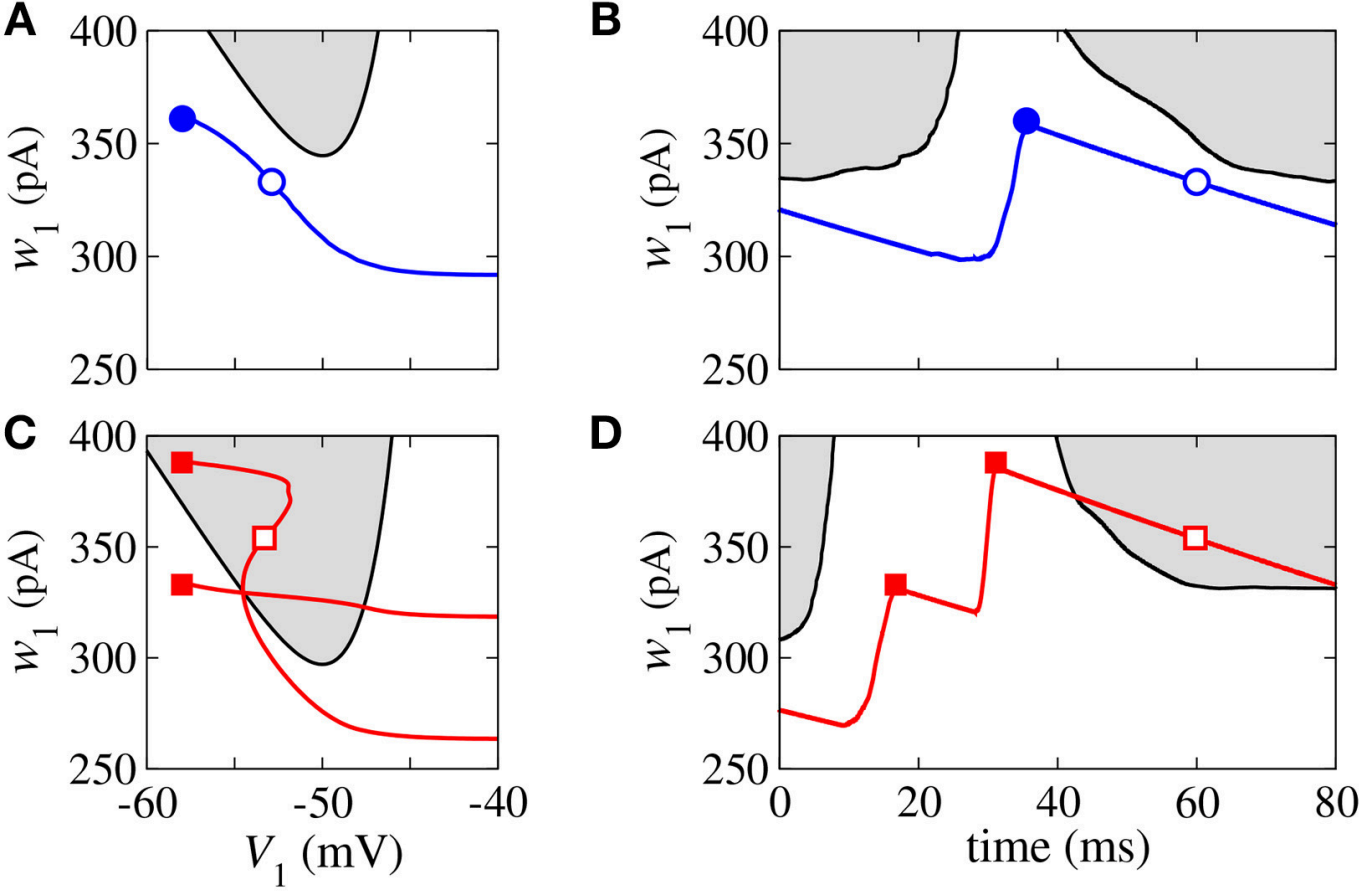
Phase space (*w*_1_, *V*_1_) **(A,C)** and time evolution of *w*_1_ **(B,D)** for spikes (blue) and burst activity (red). The gray regions correspond to *dV*_1_/*dt* < 0 and the black line represents *dV*_1_/*dt* = 0 (*V*-nullcline).

In the bistable regime, we investigate the evolution of a trajectory for a finite time interval in the phase space (*w*_*i*_, *V*_*i*_) and the time evolution of *w*_*i*_ shown in Figure 3 for *i* = 1, where the gray regions correspond to *dV*_*i*_/*dt* < 0. The boundary between the gray and white regions (black line) is given by *dV*_*i*_/*dt* = 0, the *V*_*i*_-nullcline (Naud et al., 2008). During spiking activity, the trajectory (see Figure 3A) and time evolution of *w*_*i*_ (see Figure 3B) do not cross the *V*_*i*_-nullcline. For bursting activities (see Figures 3C,D), we observe that *w*_*i*_ lies in the region enclosed by the *V*_*i*_-nullcline. The emergence of the bistable behavior is related to changes in the *V*_*i*_-nullcline caused by the variation of *I*_syn_.

### 3.3. External Square Current Pulse

Here, following a similar idea as in Antonopoulos (2016), we investigate the effect of the application of SCP on the bistable regime. We apply SCP considering different values of *A*_*I*_, *T*_*I*_, and number of removed inhibitory neurons. The SCP is immediately switched off after *T*_*I*_ and the analysis of the effect on the dynamical behavior is started.

**Figure 4:**
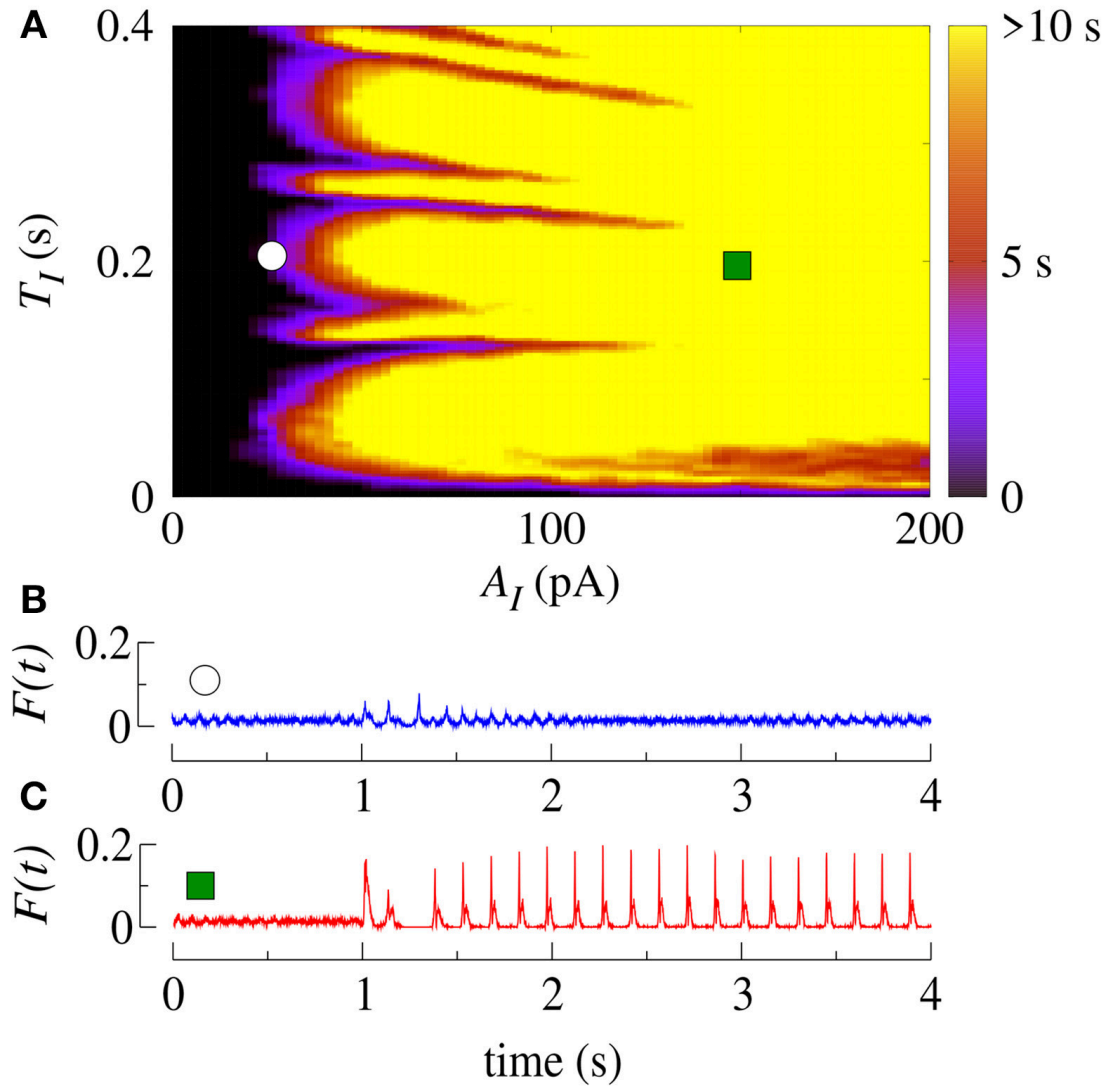
**(A)** The parameter space (*T*_*I*_, *A*_*I*_) in the bistable regime, where the color bar indicates the time the system shows synchronized burst behavior after the application of SCP. Instantaneous firing-rate for values for **(B)** white circle (*A*_*I*_ = 25pA, *T*_*I*_ = 0.2s) and **(C)** green square (*A*_*I*_ = 150pA, *T*_*I*_ = 0.2s). Note that in this figure *g*_exc_ = 0.4nS, *g* = 3 and *r* = 2.

Initially, we apply SCP to all neurons in the network with parameter values in the bistable regime with desynchronous behavior (white region in Figure 2A). Figure 4A displays the time (in color scale) that the neurons show a synchronized pattern after the application of SCP. In the black region, we see that SCP does not change the dynamical behavior, namely the neurons remain in a regime of desynchronized behavior. The yellow region depicts the values of *T*_*I*_ and *A*_*I*_ of the SCP that induce a change in the behavior of the neurons from desynchronized spikes to synchronized bursts. Picking up one point close to the border of the black and blue regions (white circle), we see that the instantaneous firing-rate (*F*(*t*)) of Equation 11 (see Figure 4B, blue line) exhibits low-amplitude oscillations corresponding to desynchronized spikes. For *T*_*I*_ and *A*_*I*_ values in the yellow region, *F*(*t*) (see Figure 4C, red line) exhibits a high-amplitude oscillation after the application of SCP, corresponding to synchronized bursts. For sufficiently large amplitudes, the change in the behavior induced by SCP does not depend on time. Importantly, perturbations with small amplitudes applied for short times is a sufficient condition for the induction of synchronous burst activity in the bistable regime. Therefore, our results suggest that even small excitatory stimuli in a random neural network arriving from other parts might be sufficient for the initiation of excessively high neural synchronization, related to the onset of epileptic seizures. Thus, further work on other neural networks that resemble brain activity might provide more insights on epileptogenesis.

**Figure 5:**
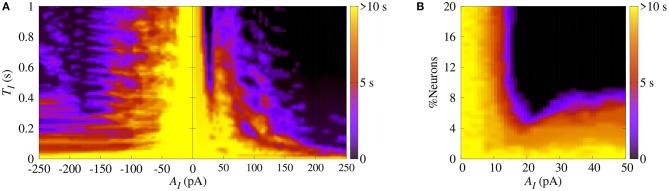
**(A)** The parameter space (*T*_*I*_, *A*_*I*_), where the color bar indicates the time the system shows synchronized burst behavior after the application of SCP. **(B)** Number of perturbed neurons as a function of *A*_*I*_. Note that in this figure we consider *g*_exc_ = 0.4nS, *g* = 3 and *r* = 2.

Similarly, we apply SCP when the neurons in the network show synchronized bursts of firing activity in the bistable regime. Here, we aim to suppressing the synchronous behavior by means of applying SCP. We consider SCP with positive and negative amplitudes applied to 10% of the neurons in the random network. Figure 5A shows how long the bursts remain synchronized after SCP is switched off (color bar). We verified that both negative and positive amplitudes exhibit regions where the synchronous behaviors are suppressed, namely there is a transition from synchronized bursts to desynchronized spikes. In addition, for *T*_*I*_>0.4 s and considering the absolute value of the amplitudes, the transition occurs for positive values with smaller amplitudes than for negative values. In Figure 5B, we show the dependence of the percentage of the perturbed neurons by the stimulus on the time the neurons remain in the bursting synchronous regime. The black region represents parameters for which the dynamics on the network does not remain synchronous, and therefore, synchronization is suppressed. In this figure, *T*_*I*_ = 1s. These results allow us to conclude that desynchronous behavior is achieved for *A*_*I*_ > 15pA and for at least 10% of the perturbed neurons.

## 4. Discussion and Conclusion

In this paper, we studied the influence of inhibitory synapses on the appearance of synchronized and desynchronized fire patterns in a random network with adaptive exponential integrate-and-fire neural dynamics. When the inhibitory influence is reduced by either decreasing the inhibitory synaptic strength or the number of inhibitory neurons, the dynamics on the network is more likely to exhibit synchronous behavior. The occurrence of synchronization results from the lack of balance between excitatory and inhibitory synaptic influences.

We found parameter values that shift to a bistable regime where the neurons can either exhibit desynchronous spiking or synchronized bursting behavior. In the bistability region, a desynchronous (synchronous) behavior becomes synchronous (desynchronous) by varying forward (backward) *g*_exc_. The onset of synchronization is thus associated with a hysteresis-loop.

We showed that, in the bistable regime, synchronized bursts can be induced by means of applying square current pulses. Our study also showed that outside the bistable regime, square current pulses do not induce synchronization. Furthermore, in the bistable regime, when neurons are synchronized, square current pulses can be used to suppress it. Positive amplitudes of square current pulses are more effective in ceasing synchronized bursts than negative ones. In addition, we showed that when one applies square current pulses to >10% of the neurons in the network, it is enough to desynchronize the dynamics. Our work shows that a decrease of inhibition contributes to the appearance of excessively high synchronization, reminiscent of the onset of epileptic seizures in the brain, thus confirming previous experimental results and theoretical models. Both decreasing the number of inhibitory neurons and the inhibitory strength, induce excessively high synchronization, related to epilepsy.

Finally, within this framework, we hypothesize that low amplitude stimuli coming from some brain regions might be capable of inducing an epileptic seizure manifested by high neural (abnormal) synchronization in other brain regions. Therefore, the work in this paper supports the common approach of the induction of square current pulses to control or treat epileptic seizures, since we have shown that such external perturbations not only can induce, but more importantly can suppress synchronous behavior in random networks with neural dynamics.

## Author Contributions

PP, FB, EL, and KI designed the work, developed the theory and performed the numerical simulations. AB wrote the manuscript with support from IC, JS, MB, CA, and JK. PJ, AK, and EM revised the manuscript for several times and gave promising suggestions. All authors contributed to manuscript revision, read and approved the submitted version.

### Conflict of Interest Statement

The authors declare that the research was conducted in the absence of any commercial or financial relationships that could be construed as a potential conflict of interest.
